# Points for Preachers, Churchworkers, and Pressmen

**Published:** 1888-06-02

**Authors:** 


### Points for Preachers, Churchworkers, and Pressmen.

### A HINT TO CHURCHWARDENS.

Mr. George P. Pocock, Churchwarden, writes from 98, Elgin Avenue, St. Peter's Park, W.:—

"Next Sunday week being Hospital Sunday, Canon Trench (who is out of town for some days) has asked me to write and ask you what would be the best way for us to advertise the fund in our church. Is the current number of The Hospital suitable for the purpose, or do you know of any better thing to use? We should like to distribute next Sunday, so I should be obliged if you would favour me with your views, and also say if you can assist us in any way."

[Yes. Our correspondent will see that he can obtain copies of this week's special number of The Hospital at five shillings per hundred for distribution at the church, and there is probably no conceivable method better adapted to fulfil Mr. Pocock's object.—Ed.]

### THE HOSPITALS' WEEK

(From MONDAY, June 4th, to SATURDAY, June 9th)

After much anxious consideration it has been decided to hold only one meeting this year during the Hospitals' Week. This meeting will take place at the Mansion House, at half-past two p.m. on Friday, June 8th. The Lord Mayor will take the chair. The Archbishop of Canterbury and the Right Hon. Henry H. Fowler, M.P., will be the principal speakers. Several of the metropolitan Members of Parliament have announced their intention of being present, and their names will be noted with considerable interest by many people resident within the metropolitan district. Tickets may be obtained on application to the secretary, Mr. Henry N. Custance, Mansion House, E.C.

The Hospitals' week movement has always been dependent for success upon the generous co-operation of the editors of the leading metropolitan papers. This co-operation has been more than cordial in the past, and this year, in lieu of the reports of meetings, space will be devoted each day, as far as possible, to hospital topics, so that no Londoner will be able to say that he has not given anything on Hospital Sunday, 1888, because he was ignorant of the great occasion, or of the pressing needs and claims of the metropolitan hospitals and dispensaries. We would offer the editors of the metropolitan daily press the most cordial acknowledgments of all who are interested in the welfare of the sick and suffering for the generous consideration and support they have extended to the metropolitan medical charities in former years, and especially for their invaluable help on the present occasion.

It will be remembered that the objects of the Hospitals' Week were to extend the interest and support already given to the hospitals and medical charities by rousing and fixing public attention to the following facts:—

(1) The pressing pecuniary necessities of these institutions; and

(2) The importance of their efficient maintenance to all classes of the community, from the highest to the lowest.

[These two objects would be secur d if everybody would send a copy of th Special Number o The Hospital to one well-to-do friend or acquaintance]

(*a*) Attending some place of worship on Hospital Sunday, June 10th, 1888.

(*b*) Sending a contribution direct to the Lord Mayor at the Mansion House, or

(*c*) Placing a donation in the boxes which will be fixed outside the Mansion House for the convenience of those who prefer to give in that way.

These objects will be admirably accomplished on the present occasion by the publicity given to the pressing needs of the hospitals by the editors of the metropolitan daily newspapers. If the clergy and ministers of religion, churchwardens and elders, will be at the trouble of distributing copies of the special number of The Hospital, which may be obtained as stated below, the nterest thus aroused could not fail to make itself felt upon the congregations when the collecting boxes are passed round on Hospital Sunday.

### TO THE CLERGY AND MINISTERS.

"The Hospital" for Distribution on Special Terms.—The Proprietors of The Hospital will send copies of this Special Supplement, on application, to any clergyman or minister of religion, who may require them for distribution amongst the more wealthy members of each congregation. They will be supplied, *if sent for by hand* to 38, Old Jewry, Cheapside, E.C., in bundles of not less than twenty-six copies, at the usual trade price, viz., one shilling and fivepence per twenty-six copies, *or five shillings per hundred copies.*

Notices of Sermons.—It is requested that an early notification may be sent to the Editor of The Hospital of all special sermons to be preached in aid of the hospitals at any of the services on Hospital Sunday, June 10th. All such notices should be addressed to the Editor of The Hospital, The Lodge, Porchester Square, W., so as to arrive there not later than Tuesday, June 5th, in time to appear in The Hospital which will be published on June 7th, in time for distribution in the churches and chapels.

